# Modulation of Voltage-Gated Sodium Channels by Activation of Tumor Necrosis Factor Receptor-1 and Receptor-2 in Small DRG Neurons of Rats

**DOI:** 10.1155/2015/124942

**Published:** 2015-10-04

**Authors:** M. Leo, S. Argalski, M. Schäfers, T. Hagenacker

**Affiliations:** Department of Neurology, University of Duisburg-Essen, Germany

## Abstract

Tumor necrosis factor- (TNF-) *α* is a proinflammatory cytokine involved in the development and maintenance of inflammatory and neuropathic pain. Its effects are mediated by two receptors, TNF receptor-1 (TNFR-1) and TNF receptor-2 (TNFR-2). These receptors play a crucial role in the sensitization of voltage-gated sodium channels (VGSCs), a key mechanism in the pathogenesis of chronic pain. Using the whole-cell patch-clamp technique, we examined the influence of TNFR-1 and TNFR-2 on VGSCs and TTX-resistant NaV1.8 channels in isolated rat dorsal root ganglion neurons by using selective TNFR agonists. The TNFR-1 agonist R32W (10 pg/mL) caused an increase in the VGSC current (*I*_Na(V)_) by 27.2 ± 5.1%, while the TNFR-2 agonist D145 (10 pg/mL) increased the current by 44.9 ± 2.6%. This effect was dose dependent. Treating isolated NaV1.8 with R32W (100 pg/mL) resulted in an increase in *I*_NaV(1.8)_ by 18.9 ± 1.6%, while treatment with D145 (100 pg/mL) increased the current by 14.5 ± 3.7%. Based on the current-voltage relationship, 10 pg of R32W or D145 led to an increase in *I*_Na(V)_ in a bell-shaped, voltage-dependent manner with a maximum effect at −30 mV. The effects of TNFR activation on VGSCs promote excitation in primary afferent neurons and this might explain the sensitization mechanisms associated with neuropathic and inflammatory pain.

## 1. Introduction

Tumor necrosis factor- (TNF-) *α* is a proinflammatory cytokine that is expressed by a variety of cell types, such as immune or neuronal cells. TNF is involved in the development and maintenance of inflammatory and neuropathic pain [1, 2]. However, the mechanisms by which TNF elicits neuropathic pain are not fully understood. The effects of TNF are mediated by two distinct receptor subtypes, TNFR-1 and TNFR-2, which colocalize in nucleated cells [3, 4]. Both TNF and its receptors are expressed in rat dorsal root ganglion (DRG) neurons and are upregulated after nerve injury [5, 6].* In vivo* application of TNF to DRG neurons induces pain-related behavior in rats [6], which is accompanied by mechanical and thermal hyperalgesia. These pain-inducing effects are prevented by preemptively using TNF-neutralizing agents [7, 8] or by inhibiting the TNF-signaling pathway [6]. In addition, TNF influences neuronal excitability by increasing voltage-gated sodium channel (VGSC) currents (*I*_Na(V)_), which promotes action potential generation and may maintain neuropathic pain [9]. Local application of TNF to nociceptive neurons evokes action potentials and increases discharge rates of nerve fibers [10]. Tetrodotoxin- (TTX-) resistant NaV1.8 channel currents (*I*_NaV(1.8)_), which are essential in the pathogenesis of neuropathic pain, are increased by a TNF-mediated MAP kinase-dependent pathway in DRG neurons [11, 12]. Furthermore, the lack of TNFR-1 in TNFR-1^−/−^ mice led to reduced mechanical hypersensitivity, which is induced by exogenous TNF or inflammation [13]. Neutralizing antibodies against TNFR-1 also reduced thermal or mechanical hypersensitivity induced by nerve injury, while antibodies against TNFR-2 were ineffective [14]. These results suggest a crucial role for TNFR-1, but not TNFR-2, in the sensitization of VGSCs [12].

In the present study, we examined the influence of TNFR-1 and TNFR-2 on the modulation of *I*_Na(V)_ and *I*_NaV(1.8)_ in rat DRG neurons by using selective TNFR agonists.

## 2. Methods

### 2.1. Animals

Adult male Wistar rats (3 weeks old, 80–120 g) were used. All experiments were performed in accordance with the guidelines of the Animal Care and Use Committees of the University of Duisburg-Essen, Germany. All animals were kept on a 14/10 h light/dark cycle with water and food pellets available* ad libitum*.

### 2.2. Selective TNF Receptor Agonists

TNF mutant proteins were used (given by the P. Vandenabeele Lab, Ghent, Belgium) for the selective stimulation of either TNFR-1 or TNFR-2. The R32W and D145 proteins contain double mutations (R32W/S86T and D143N/A145R, resp., [15, 16]) and can selectively activate rat TNFR-1 and TNFR-2, respectively [17]. Mutated TNF proteins R32W and D145 have been extensively tested and showed differential binding to purified TNF receptors. The binding studies have been confirmed by biological assays using either TNFR-1 or TNFR-2 [16]. Each receptor-specific protein was dissolved in an ACSF vehicle containing 0.1% bovine serum albumin (BSA).

### 2.3. Cell Culture

DRG neurons were isolated from 3-week-old Wistar rats. The animals were anaesthetized with isoflurane. In the absence of pain reflexes, the animals were decapitated. The spinal column was removed and opened from the dorsal side. After dissection of the spinal cord, the DRGs were collected and placed in ice cold F12 media (Biochrom AG, Germany). Under optic control, the spinal nerves were cut off and the ganglion capsules were opened. The capsules were transferred into a medium containing 0.9 mL of F12 and 0.1 mL of collagenase (2612.5 U/mL, Type II, Biochrom AG, Germany) and incubated for 45 min in a humidified atmosphere containing 5% CO_2_ at a temperature of 37°C. To remove the collagenase, the DRGs were washed three times in 1 mL of F12 medium. Afterwards, the DRGs were trypsinized (2525 U trypsin/mL F12 medium) for 2 min under the same conditions. DRGs were washed twice, left in a final volume of 0.7 mL of F12 medium, and triturated with a pipette until the neurons were released. A total of 50 *μ*L of this suspension was placed in the middle of each Petri dish (3 cm, Falcon Easy Grip). Cells were incubated for at least 2 h so that the neurons could adhere to the dish, and then 1 mL of medium containing F12 and 10% horse serum (Biochrom AG, Germany) was added to each dish.

### 2.4. Electrophysiology

*I*_Na(V)_ and *I*_NaV(1.8)_ were isolated by performing the whole-cell patch-clamp technique using HEKA EPC 10 amplifier with Patchmaster software (HEKA Electronics, Germany). Only cells with a small diameter < 30 *μ*m were chosen. Microelectrodes, consisting of borosilicate glass (Biomedical Instruments), were pulled with a HEKA Pipette Puller (PIP6, HEKA Electronics, Germany) and were fire polished to a final resistance of 4-5 MΩ by using a microforge (Narishige, Japan). This relatively high resistance was intentionally chosen to guarantee the necessary stable configuration during the course of the experiments. Although it is possible that this configuration can have a negative effect on the electrodes' ability to pass sufficient current, especially at peak currents while maintaining a current-voltage relationship (*IV*-curve), this configuration allowed us to analyze the underlying mechanisms that have small effects on the current.

Before starting experiments, F12 culture medium was replaced by an external solution containing 72 mM NaCl, 72 mM choline-Cl, 2.5 mM KCl, 10 mM HEPES, 10 mM glucose, and 50 *μ*M CdCl and adjusted to pH of 7.4 with NaOH. The internal pipette solution contained 140 mM CsCl, 5 mM NaCl, 10 mM HEPES, 10 MM EGTA, and 4 mM MgCl2 and was adjusted to pH of 7.2 with TEA-OH.

The* IV*-curve for *I*_Na(V)_ was recorded for depolarizing steps starting at −60 mV and increased stepwise by 10 mV to maximum depolarization of +60 mV after hyperpolarizing prepulse to −120 mV for 500 ms to recover sodium channels from state of inactivation. Application of the drugs started after obtaining 2* IV*-curves under control conditions. For time course experiments, TNFR agonists were applied after 10 control depolarization procedures. NaV1.8 currents were isolated after eliminating tetrodotoxin-sensitive currents using 500 nM tetrodotoxin in the external solution and maintaining a −80 mV potential, which efficiently eliminates the persistent tetrodotoxin-resistant NaV1.9 current as shown before [18, 19].* IV*-curves of isolated NaV1.8 currents were similar to other curves described elsewhere [20]. The somata of the small DRG neurons were classified by their diameters (15~30 *μ*m) and *C*_*m*_ (≤45 pF). Neurons were not considered for analysis if they had high leakage currents (holding current > 1.0 nA at −80 mV), membrane blebs, total sodium current < 500 pA, or access resistance > 5 MΩ. Access resistance was monitored throughout the experiment and data were not used if resistance changes of >20% occurred. The offset potential was zeroed before patching the cells and checked after each recording for drift. Data were sampled at 10 kHz, compensated for series resistance, and stored on hard disk.

### 2.5. Data Analysis

All currents were online corrected by using a P/4 protocol. All *I*_Na(V)_ used for the time course and current-voltage relationships were rundown corrected assuming linear rundown. Current values were standardized to the mean current before application of the drugs (= 100%). For calculation of the mean current, 20 data points were used. The nonresponders were given a value of 0 in the calculation determining the current increase.

### 2.6. Statistical Analysis

All data are given as the mean +/− standard deviation. Data were analyzed by using double-sided Student's *t*-test. A difference was accepted as significant if *p* < 0.05.

## 3. Results

**Figure 1:**
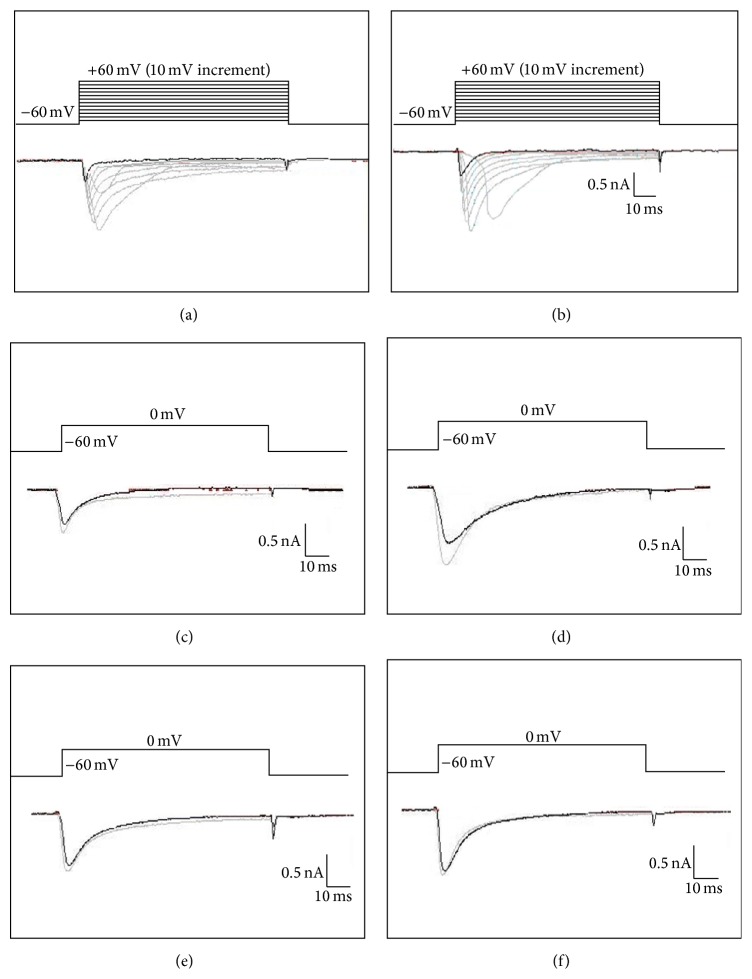
Representative traces of VGSCs. Cells were depolarized to a variety of membrane potentials ((a) *I*_Na(V)_ and (b) *I*_Na(V1.8)_). *I*_Na(V)_ currents before (black) and after (gray) application of 10 pg R32W (c) and D145 (d). *I*_Na(V1.8)_ currents before (black) and after (gray) application of 100 pg R32W (e) and D145 (f).

*I*_Na(V)_ was successfully recorded from 112 DRG neurons. Cells were classified as responders if the current was changed by at least 10% (*n* = 71). Depolarization of DRG neurons from the holding potential to 0 mV led to an inwardly directed, inactivating current. Administration of the dissolving agents (ACSF + 0.1% BSA) does not change the currents (data not shown). Treatment of DRG neurons with either R32W or D145 led to an increase of *I*_Na(V)_ and *I*_NaV(1.8)_ (Figures 1(a) and 1(b)).

**Figure 2:**
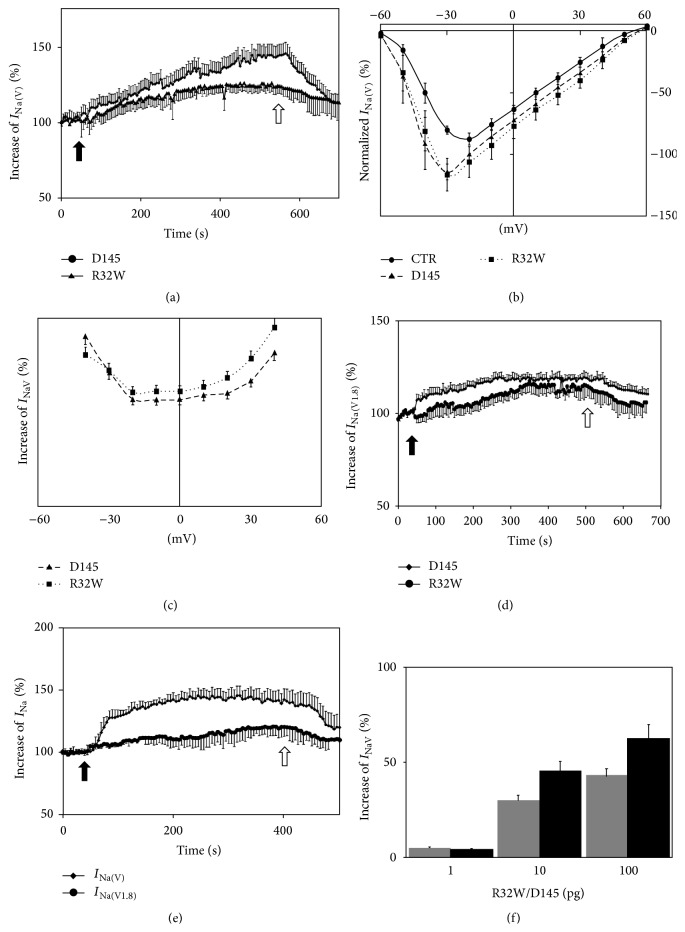
Characterization of R32W and D145 effects on *I*_Na(V)_ and *I*_Na(V1.8)_ currents. (a) Time course of normalized *I*_Na(V)_ during repetitive depolarization from the holding potential to 0 mV before and after application of 10 pg R32W or 10 pg D145 (black arrow: time of application; white arrow: washout). (b) *IV*-curve of *I*_Na(V)_ (black: control conditions; speckled line: after application of 10 pg R32W; dashed line: after application of 10 pg D145). Cells were depolarized to a variety of potentials (−60 to 60 mV) from a holding potential of –80 mV at increments of 10 mV to elicit *I*_Na(V)_. (c) Voltage-dependent reduction of *I*_Na(V)_ after administration of 10 pg R32W or 10 pg D145. (d) Time course of normalized *I*_Na(V1.8)_ during repetitive depolarization from the holding potential to 0 mV before and after application of 100 pg R32W or 100 pg D145 (black arrow: time of application; white arrow: washout). (e) Time course of normalized *I*_Na(V)_ and *I*_Na(V1.8)_ during repetitive depolarization from the holding potential to 0 mV before and after application of 10 pg R32W* and* 10 pg D145 (black arrow: time of application; white arrow: washout). (f) R32W and D145 dose-dependent increase in *I*_Na(V)_.

Application of R32W (10 pg) led to an increase of *I*_Na(V)_ by 27.2 ± 5.1%. A steady state current was reached after 415 s (*n* = 11). Application of D145 (10 pg) increased *I*_Na(V)_ by 44.9 ± 2.6%, reaching a steady state after 500 s (*n* = 8). During the washout, the currents return to almost baseline (111.45 ± 8.5% versus 112.87 ± 7.6%) (Figure 2(a)).

In the* IV*-curve, a maximum current was elicited at depolarization to −30 mV. R32W and D145 increased the current in a bell-shaped voltage-dependent manner in the range between −50 mV and +60 mV (*n* = 10 each). Reversal potentials were at +50 mV and were not changed by the application of D145 or R32W (Figures 2(b) and 2(c)).

During repetitive depolarization procedures to 0 mV, application of either R32W (100 pg) (*n* = 8) or D145 (100 pg) (*n* = 8) led to an increase in *I*_NaV(1.8)_ by 18.9 ± 1.6% and 14.5 ± 3.7% 300 s after treatment, respectively (Figure 2(d)).

The dual application of R32W and D145 (100 pg) led to an increase of *I*_Na(V)_ by 45.2 ± 7.4% 320 s after treatment (*n* = 7), while *I*_NaV(1.8)_ was increased by 20.6 ± 6.8% 400 s after application (*n* = 7) during repetitive depolarization to 0 mV (Figure 2(e)).

Using increasing concentrations of R32W or D145 (1–100 pg) led to an increase of *I*_Na(V)_ in a dose-dependent manner (Figure 2(f)).

**Figure 3:**
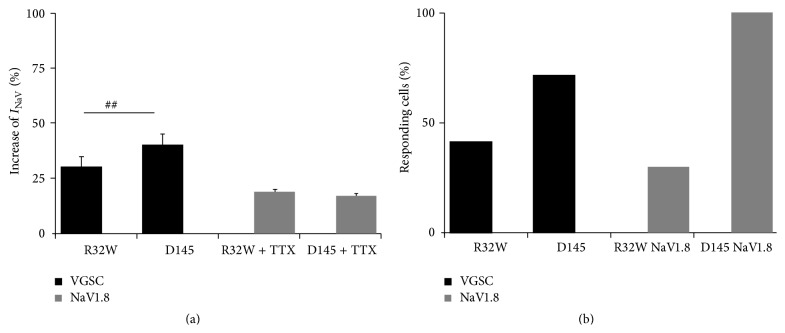
(a) Increase in *I*_Na(V)_ (10 pg of each drug) and *I*_Na(V1.8)_ (100 pg of each drug) currents after application of R32W or D145 during repetitive depolarization to 0 mV after 600 s when the current reaches a steady state (^##^ *p* < 0.01). (b) Amount of responding cells, including *I*_Na(V)_ or *I*_Na(V1.8)_, in the cell population. Cells were classified as responding cells if the currents were affected by more than 10%.

Application of D145 (10 pg) led to significantly higher increase of *I*_Na(V)_ compared to the application of R32W (10 pg). Treatment with either 100 pg of D145 or R32W led to a significantly higher increase of *I*_Na(V)_ compared to *I*_NaV(1.8)_ (Figure 3(a)). In both settings, when measuring isolated *I*_Na(V)_ and *I*_NaV(1.8)_, the amount of responding cells was higher using D145 than R32W (Figure 3(b)).

## 4. Discussion

This study presents evidence for differential modulation of VGSC and the TTX-resistant subtype NaV1.8 by TNFR in small DRG neurons. TNF has been shown to regulate a variety of ion channels. It decreases potassium channel currents in retinal ganglion neurons [21] and increases calcium channel currents in hippocampal and cultured superior cervical ganglion neurons [22, 23]. In DRG neurons, a decrease of voltage-gated calcium channel currents and an increase in *I*_Na(V)_ have been described, while voltage-gated potassium channel currents were not affected, suggesting that TNF has differential effects depending on the ion channel and cell type [9]. An increase in *I*_Na(V)_ may promote hyperexcitability, which is a key symptom of neuropathic pain. Besides the long-lasting effects of TNF by regulating the expression of a variety of inflammatory mediators and modifying signaling proteins, the application of TNF has rapid onset effects, which suggest interactions with primary excitation proteins such as VGSC. The enhancement of TTX-resistant VGSC currents starts in <60 s and the enhancement of VGSC currents in DRG neurons begins within 20 s after TNF administration [9, 12, 24]. Using* in vitro* single-fiber recordings for isolating A*δ*- and C-fiber activity in DRG neurons, perfusion with TNF increases rapid firing rates, also suggesting a direct interaction between VGSCs and TNF [25]. Variations in TNF responses exist but can be explained by technical reasons, that is, distance from application pipette to the neuron. Interestingly, the increase in *I*_Na(V)_ is higher when using selective agonists compared to the use of TNF alone. In other systems, TNF has been shown to reduce *I*_Na(V)_ via a PKC-dependent pathway, which may counteract effects on VGSC via other signaling pathways [26]. This can explain why TNF mediates different effects compared to the selective agonists alone. The application of TNF to DRG neurons induces mechanical allodynia and mechanical sensitivity of C-fibers [27, 28]. These results suggest an important role for the interaction of TNF and VGSC.

TNF promotes its effects via the constitutively expressed TNFR-1 and the inducible TNFR-2. Different intracellular signal pathways are affected by TNFR-1 and TNFR-2 activation. While TNFR-1 activation leads to internalization of the receptor, TNFR-2 activation is followed by shedding of the ligand-receptor complex [29]. Consequently, TNFR activation elicits distinct effects. For example, it has been shown that activation of TNFR-1 by local application of TNF to naïve DRGs induced high-frequency firing of A*β*- and A*δ*-fibers, while TNFR-2 activation had no effect. In contrast, after nerve injury, both TNFR-1 and TNFR-2 activation increased discharge rates [25]. TNFR-1 seems to be very important in the pain-sensitizing actions of TNF. Mechanical hypersensitivity induced by inflammation or nerve injury is reduced in TNFR-1 knockout mice [13]. In addition, neutralizing antibodies against TNFR-1 reduced pain-associated behavior, while antibodies against TNFR-2 were noneffective [14]. These results underlie the different roles of TNFR-1 and TNFR-2 in pathologic conditions and offer the possibility to target neuropathic pain.

In contrast to previous studies, our results showing that there is a larger increase of *I*_Na(V)_ after activation of TNFR-2 and an increase in reacting cells provide evidence that TNFR-2 has a more important role than TNFR-1 in the modulation of nerve excitability. The higher rate of responding cells when isolating *I*_NaV(1.8)_ is surprising. A possible reason for this higher rate may be that the membrane-bound receptors are coexpressed. It is possible that the activation of TNFR-1/TNFR-2 leads to higher response rates when coexpressed with NaV1.8 in comparison to neurons that coexpress TNFRs with other isoforms of NaV. The coexpression of TNFR and NaV isoforms has been shown for NaV1.7 in DRG neurons and chromaffin cells [30]. In addition, the expression of TNFR-2 has been shown to be closely related to NaV1.7 and NaV1.8 expression in sensory neurons, which may explain the predominant responding rates of D145 compared to R32W [31]. This effect may be important in the initial phase of inflammatory and neuropathic pain but may be superimposed by the changes in the pattern of expression of TNFR-1 and TNFR-2 after nerve injury and explain the effects of TNFR-1 and TNFR-2 seen in other studies, especially after nerve injury [6, 25]. The shift in the* IV*-curve and the increase of *I*_Na(V)_ in the voltage above 0 mV may be explained by an increase of the available channels or an increase in channel permeability.

Several intracellular pathways have to be examined for downstream signaling of TNFR activation. VGSCs have various phosphorylation sites, on which modification may lead to changes in the biophysical properties of channel gating. Recently, chelerythrine has been shown to suppress the inhibitory effect of TNF on sodium channel currents of skeletal muscle cells, suggesting the involvement of protein kinase C (PKC) in regulation of VGSC [26, 32]. In addition, it is known that modulation of VGSCs is dependent on the special channel subtype [33]. It has been shown that the activation of TNFR-1 increases *I*_NaV(1.8)_ rapidly in mouse DRG neurons by p38-dependent mechanisms [12]. p38 may enhance *I*_NaV(1.8)_ by phosphorylating the NaV1.8 channel or an associated protein. In addition, p38 has been shown to directly modulate other voltage-gated channels [34]. In inflammatory responses, NF-*κ*B has been shown to be involved in TNF-mediated ion channel regulation [35]. Pathways with common final targets are possible due to the observation that the dual application of TNFR agonists has no additive effect on *I*_Na(V)_ or *I*_NaV(1.8)_. The possibility of TNFR-subtype dependent signaling pathways will have to be addressed in further studies.

The limitations of our study need to be discussed. Because the mutant peptides were designed to target human TNFR, we cannot exclude the possibility that there are different effects in rat DRGs. However, the cross-reactivity of our mutants has been established before using a cell death assay [36]. The efficacy of the mutants may differ in TNFR-1 and TNFR-2. Our study only uses naïve DRG neurons. Because of the several pathways activated after nerve injury, our results concerning TNF as well as other inflammatory mediators or posttranslational modification of ion channel activity may be limited by long-lasting superimposing effects. We only used* in vitro* techniques; hence, the results may translate to* in vivo* effects. Another aspect to consider is the expression rate of TNFR-1 and TNFR-2 in nociceptive neurons. In our study, we found a responding rate of ~60% in all experiments. Other studies of mouse and rat DRG have shown that TNFR-1 or TRPV1 is expressed in up to 40% of the sensory neurons. Most of the TRPV1-expressing neurons coexpress isolectin-B4. While >30% of these neurons coexpress TNFR-1 and TRPV1, only 10% of the neurons coexpress both TNFR and IB4 [37]. In small sized DRG neurons (~30 *μ*m), TRPV1 is expressed in ~50% of the cell population [38]. To our knowledge, the coexpression rates of TNFR-2 and TRPV1 or IB4 have not been described. This may explain the differential effects of TNFR agonists in different cell populations. Further experiments have be designed to determine whether this is the case.

In conclusion, our study provides evidence for a potential role of TNFR-2 in the generation of hyperexcitability by increasing of VGSC currents in uninjured (or peracute injured) neurons, which may be a relevant mechanism in neuropathic and inflammatory pain conditions.
